# In vitro activity and rodent efficacy of clinafloxacin for bovine and swine respiratory disease

**DOI:** 10.3389/fmicb.2013.00154

**Published:** 2013-06-14

**Authors:** Michael T. Sweeney, Rebecca Quesnell, Raksha Tiwari, Mary LeMay, Jeffrey L. Watts

**Affiliations:** Global Therapeutics ResearchZoetis, Kalamazoo, MI, USA

**Keywords:** clinafloxacin, respiratory diseases, minimum inhibitory concentration, efficacy, mouse models, veterinary medicine

## Abstract

Clinafloxacin is a broad-spectrum fluoroquinolone that was originally developed and subsequently abandoned in the late 1990s as a human health antibiotic for respiratory diseases. The purpose of this study was to investigate the activity of clinafloxacin as a possible treatment for respiratory disease in cattle and pigs. Minimum inhibitory concentration (MIC) values were determined using Clinical and Laboratory Standards Institute recommended procedures with recent strains from the Zoetis culture collection. Rodent efficacy was determined in CD-1 mice infected systemically or intranasally with bovine *Mannheimia haemolytica* or *Pasteurella multocida*, or swine *Actinobacillus pleuropneumoniae*, and administered clinafloxacin for determination of ED_50_ (efficacious dose-50%) values. The MIC_90_ values for clinafloxacin against bovine *P. multocida*, *M. haemolytica*, *Histophilus somni*, and *M. bovis* were 0.125, 0.5, 0.125, and 1 μg/ml, respectively, and the MIC_90_ values against swine *P. multocida*, *A. pleuropneumoniae*, *S. suis*, and *M. hyopneumoniae* were í0.03, í0.03, 0.125, and í0.008 μg/ml, respectively. Efficacy in mouse models showed average ED_50_ values of 0.019 mg/kg/dose in the bovine *M. haemolytica* systemic infection model, 0.55 mg/kg in the bovine *P. multocida* intranasal lung challenge model, 0.08 mg/kg/dose in the bovine *P. multocida* systemic infection model, and 0.7 mg/kg/dose in the swine *A. pleuropneumoniae* systemic infection model. Clinafloxacin shows good *in vitro* activity and efficacy in mouse models and may be a novel treatment alternative for the treatment of respiratory disease in cattle and pigs.

## INTRODUCTION

Respiratory disease in cattle and pigs is one of the most economically important diseases of the feedlot industry with global losses estimated to be over $3 billion per year (Watts and Sweeney, 2010). Respiratory disease in these livestock animals is a multi-factorial and multi-agent disease. Transportation over long distances, often associated with exhaustion, starvation, dehydration, chilling, or overheating depending on weather conditions, dusty environmental conditions in the feedlot, and nutritional stress associated with changes in the diet all serve as stressors leading to “shipping fever” (). Additionally, viral infections are important etiological agents for subsequent bacterial infections in which *Mannheimia haemolytica*, *Pasteurella multocida*, *Histophilus somni*, and *Actinobacillus pleuropneumoniae* are the most important pathogens (Watts and Sweeney, 2010). The fluoroquinolone antimicrobial agents danofloxacin, enrofloxacin, and marbofloxacin are currently approved for the treatment of bovine respiratory disease (BRD) and swine respiratory disease (SRD) caused by these bacterial pathogens. There may be a need for newer antimicrobial agents with unique mechanisms of action due to continued emergence of bacterial resistance to danofloxacin, enrofloxacin, and marbofloxacin by these pathogens (Watts and Sweeney, 2010).

Clinafloxacin was first introduced into the literature in 1991 and was reported to have excellent broad *in vitro* activity against medically significant bacteria (Cohen et al., 1991). This compound was developed as a human health agent for the treatment of bacterial pneumonia but subsequently abandoned due to specific adverse events related to hypoglycemia and photosensitivity (Siami et al., 2002; Chow, 2003). Due to the extensive toxicological safety package, this compound was re-evaluated as an animal health antimicrobial agent for the treatment of bacterial infections that cause respiratory disease in cattle and pigs. The purpose of this study was to determine the *in vitro* activity and rodent efficacy of clinafloxacin when tested against pathogens associated with BRD and SRD.

## MATERIALS AND METHODS

### BACTERIAL STRAINS

Bacterial strains from bovine and swine respiratory infections were used in the present study and came from the Zoetis surveillance program isolated from clinical cases of BRD and SRD. Bovine isolates used in the studies (*P. multocida*, *M. haemolytica*, *H. somni*) were part of the 2008 Zoetis surveillance program and swine isolates (*P. multocida*, *A. pleuropneumoniae*, *S. suis*) were part of the 2009 surveillance program. Additionally, mycoplasmas were recovered from tulathromycin clinical trials from 2008 to 2010. All isolates were identified by clinical diagnostic laboratories using biochemical methods. The isolates were frozen in trypticase soy broth containing 10% glycerol and maintained at -70°C as part of the Zoetis culture collection. Isolates were plated and grown on trypticase soy agar plates containing 5% sheep blood. The quality control strains *Staphylococcus aureus* ATCC 29213, *Enterococcus faecalis* ATCC 29212, *Escherichia coli* ATCC 25922, *Streptococcus pneumoniae* ATCC 49619, and *Mannheimia haemolytica* ATCC 33396 were originally acquired from the American Type Culture Collection (Manassas, VA, USA).

### ANTIMICROBIAL AGENTS

The antibiotic powders used in the experiments were obtained from the indicated sources: danofloxacin, enrofloxacin (Riedel-de Haen); clinafloxacin, ceftiofur (Zoetis).

### ANTIMICROBIAL SUSCEPTIBILITY TESTING

Cation-adjusted Müller-Hinton broth (MHB; Difco Laboratories, Detroit, MI, USA) was used for susceptibility testing of *P. multocida* and *M. haemolytica*. Cation-adjusted MHB supplemented with 2.5% lysed horse blood was used for susceptibility testing of *S. suis*. Veterinary fastidious medium (VFM) broth (TREK Diagnostic Systems) was used for susceptibility testing of *A. pleuropneumoniae* and *H. somni*. The minimum inhibitory concentration (MIC) values were determined using the broth microdilution protocol according to the standards of the [Clinical and Laboratory Standards Institute (CLSI), 2008]. The MIC was the lowest concentration of drug which prevented macroscopically visible growth under the conditions of the test. Assay control was monitored with ATCC quality control strains. MICs were done in duplicate on two separate days. When MICs for all compounds tested were within one doubling dilution, the original results were used for analysis and reporting. On those occasions where results differed by more than one doubling dilution, the isolate was tested a third time and those results were used for analysis unless the results were inconsistent with either of the previous two sets of MIC results. When an isolate tested differently all three times it was determined to be too inconsistent in testing to be included in the analysis. In those rare instances, a new bacterial isolate was selected for MIC testing. Determination of MICs against all mycoplasma isolates were conducted at Microbial Research, Inc. (MRI) in Fort Collins, CO, USA. There is no standardized procedure for the susceptibility testing of veterinary *Mycoplasma* listed in CLSI document M31-A3 (CLSI, 2008). MIC plates were prepared by MRI using Modified Hayflick’s broth with alamarBlue for isolates except *M. hyopneumoniae *which required Friis broth. Each well contained 50 μl of the appropriate diluted drug prepared at 2× the final concentration and each well was inoculated with 50 μl of each isolate. Plates were incubated aerobically at 36°C for 16–24 h (*M. bovis* and *bovirhinis*) and 2–14 days (*M. hyopneumoniae*). There are no published quality control ranges available for the testing of *Mycoplasma* species, however, *M. hyopneumoniae* ATCC 25934 was included as part of the test isolates. Positive growth control wells for each tested isolate were monitored and sterility checks of plates by sterile diluent inoculation of the negative control well was performed. MIC results were the lowest concentration of antimicrobial agent that completely inhibited growth of the organism as detected by color change of the broth when color change was noted in the positive control well.

### TIME-KILL ASSAYS

The bactericidal effects of clinafloxacin and comparator fluoroquinolones against 10 bacterial isolates were assessed by a time-kill assay. For *M. haemolytica* and *P. multocida*, cultures were grown overnight at 35°C on Müller-Hinton agar and for *H. somni* and *A. pleuropneumoniae* cultures were grown on chocolate Müller-Hinton agar. Assay tubes were prepared to contain 8.5 ml of the appropriate medium (cation-adjusted MHB for *M. haemolytica* and *P. multocida*, and VFM for *H. somni* and *A. pleuropneumoniae*), 0.5 ml of the antibacterial agent, and 1.0 ml of the McFarland-equivalent culture in appropriate medium which resulted in a cell concentration of approximately 10^7^ CFU/ml for most cultures. The final drug concentrations tested were 1× and 4× the MIC for each test strain. An identical assay tube that contained broth and inoculum, but no antibacterial agent, served as the culture growth control. The assay tubes were incubated at 35°C and samples were removed for viable counts at 0, 2, 6, and 24 h for all cultures. A 100-μl sample was removed from the assay tube at each timepoint and serial dilutions (10^-^^1^ to 10^-^^8^) were prepared in appropriate media. A 100-μl volume of each dilution was applied to duplicate blood agar plates (BAPs), spread evenly over the plate surface, and incubated overnight at 35°C with or without CO_2_. Colonies were manually counted and plates at a dilution yielding a count of 20–200 were utilized.

### MPC DETERMINATIONS

Methods for mutant prevention concentrations (MPCs) were determined following the method of Blondeau et al. (2001). Briefly, two bacterial strains, *S. suis* and *P. multocida* from SRD and BRD, respectively, were selected for testing and subcultured to multiple agar plates (five each) and incubated for 18–24 h under proper incubating conditions for the microorganisms. The contents of the inoculated agar plates were removed with a sterile swab and transferred to cation-adjusted MHB or cation-adjusted MHB containing 2.5% lysed horse blood (250 ml). The inoculated broth was incubated for 18–24 h under proper incubating conditions. Broth cultures were centrifuged and the pellet resuspended in a lower volume of fresh broth media. Once the bacterial density was adjusted, d10^9^ CFUs were inoculated to agar plates containing clinafloxacin, danofloxacin, or enrofloxacin at twofold concentrations ranging from 256 to 0.007 μg/ml and incubated under ambient conditions at 35°C. Cultures were read at 24 and 48 h and the lowest drug concentration that prevented growth was determined as the MPC. Based on parent MIC, an MPC/MIC ratio was determined for evaluation. Data for time-kill and MPC analyses were collected once and results were consistent with data from previous journal reports.

### MOUSE EFFICACY ASSAYS

Female CD-1 mice were acquired from Charles River Labs (Portage, MI, USA) at a weight of 13–14 g, and acclimated for 3–6 days. Mice were randomized when delivered on-site and housed 10/large shoebox cage, on litter with nestlets for environmental enrichment. On Study Day 1, approximately 10% of the mice were weighed to provide an average weight for calculation of drug concentrations to be administered. On Study Day 0, all mice received a challenge inoculation (induced infection). Compounds tested included clinafloxacin, enrofloxacin, and ceftiofur. All mice received one to three subcutaneous (SC) injections of stated compounds starting at 1 or 18 h post-challenge (PC) depending on the model. SC injections were administered in the left and/or right inguinal region, using a 28 gage X½ inch insulin needle-syringe to deliver 0.1 ml per injection. Serial two- or threefold dilutions of test compounds were dosed to different groups of mice, and mice were monitored for 6 or 8 days. Efficacy of test compounds was calculated as ED_50_ values (efficacious dose-50%) by use of GraphPad Prism^®^. All animal studies were conducted under the guidelines, and with the approval of, the Pfizer Animal Health Institutional Animal Care and Use Committee.

For the *Mannheimia haemolytica* systemic infection model, 25 colonies from overnight growth on BAP were aseptically picked and inoculated into 50 ml of room temperature brain-heart infusion broth (BHIB; BBL^TM^, Becton, Dickinson and Company) and placed at 35–37°C for 3.5–4 h of incubation. Appropriate dilutions in cold BHIB with 4% brewer’s yeast were made to achieve ~2.5 × 10^8^ CFU/ml. A volume of 0.2 ml of this adjusted challenge material was administered to mice by the intraperitoneal (IP) route using a 25 gage X 5/8 inch needle on a 1-ml tuberculin syringe. Placement of the IP challenge was in the mouse’s lower right abdominal quadrant. Drugs were administered via SC injection as described above at either 1 and 3 h PC or at 1, 24, and 48 h PC. Mice were monitored for 6 days.

For the *Pasteurella multocida* intranasal (IN) infection model, five colonies from the overnight growth on BAP were picked and inoculated into 10 ml of room temperature BHIB. Appropriate dilutions in cold BHIB were made to achieve ~1.25 × 10^5^ CFU/ml. Mice were anesthetized either by an IP injection of 100 mg/kg ketamine + 10 mg/kg xylazine, or by isoflurane inhalation via the Compac 5 Anesthesia Center. A volume of 0.04 ml (40 μl) of the adjusted challenge material was slowly applied to the nares of anesthetized mice, using a Rainin L100 Pipet Lite manual pipettor with sterile tips. At 18 h PC, mice received one SC injection of 0.1 ml of appropriate dilutions of drug prepared in phosphate buffered saline (PBS). Mice were monitored for 8 days.

For the *Pasteurella multocida* systemic infection model, dilutions in cold PBS were made to achieve ~1.5 × 10^4^ CFU/ml. A volume of 0.1 ml of this adjusted challenge material was administered to mice by the IP route as described above. Drugs to be tested were administered via SC injection as described above at 1 and 3 h PC. Mice were monitored for 6 days.

For the *A. pleuropneumoniae* systemic infection model, fresh supplement C at 2% concentration was added to 30 ml of room temperature BHIB. Fifteen *A. pleuropneumoniae* colonies were transferred to the BHIB with supplement C, then incubated for ~4 h until the A_600_ of undiluted growth approximated a pre-determined optical density known to yield ~2 × 10^8^ CFU/ml. Mice were challenged with a volume of 0.2 ml/mouse, by IP administration, as described above. Drugs to be tested were administered via SC injection as described above at 1, 24, and 48 h PC. Mice were monitored for 6 days.

## RESULTS

### ANTIMICROBIAL SUSCEPTIBILITY TESTING

The MICs of clinafloxacin and comparator compounds for 480 bacterial clinical isolates are summarized in **Table 1**. Clinafloxacin demonstrated strong activity against all tested bovine and swine isolates with MIC_90_ values lower for all isolate groups compared to danofloxacin and enrofloxacin (MIC_90_ values bovine *P. multocida*, *M. haemolytica*, *H. somni*, and *M. bovis* were 0.125, 0.5, 0.125, and 1 μg/ml, respectively, and the MIC_90_ values against swine *P. multocida*, *A. pleuropneumoniae*, *S. suis*, and *M. hyopneumoniae* were ≤0.03, ≤0.03, 0.125, and ≤0.008 μg/ml, respectively). Clinafloxacin was also very active against mycoplasma isolates recovered from cattle and pigs. Clinafloxacin MICs obtained against ATCC cultures in this study were similar to what is listed as acceptable QC parameters in CLSI (formerly National Committee for Clinical Laboratory Standards, NCCLS; CLSI, 2005; data not shown).

**Table 1:** *In vitro* MIC determinations of clinafloxacin and comparator drugs.

Organism (no. of strains)	Antimicrobial agent	MIC (μg/ml)		
		Range	50%	90%
*P. multocida* (90)^a^	Clinafloxacin	0.003–0.25	0.007	0.125
	Danofloxacin	0.007–8	0.03	2
	Enrofloxacin	0.007–8	0.03	2
*M. haemolytica* (95)^a^	Clinafloxacin	0.003–0.5	0.007	0.5
	Danofloxacin	0.007–16	0.03	16
	Enrofloxacin	0.007–16	0.03	16
*H. somni* (97)^a^	Clinafloxacin	0.003–2	0.007	0.125
	Danofloxacin	0.03 to >16	0.06	4
	Enrofloxacin	0.015 to >16	0.03	2
*P. multocida* (60)^b^	Clinafloxacin	0.015–0.06	0.015	0.015
	Danofloxacin	0.015–0.25	0.06	0.125
	Enrofloxacin	0.015–0.12	0.06	0.06
*A. pleuropneumoniae* (50)^b^	Clinafloxacin	0.015–0.06	0.015	0.015
	Danofloxacin	0.015–0.25	0.06	0.125
	Enrofloxacin	0.015–0.12	0.015	0.06
*S. suis* (50)^b^	Clinafloxacin	0.015–1	0.06	0.125
	Danofloxacin	0.25–8	0.5	1
	Enrofloxacin	0.125–4	0.5	1
*M. bovis* (10)^b^	Clinafloxacin	0.03–1	0.03	1
	Danofloxacin	0.125–2	0.5	2
	Enrofloxacin	0.25 to >8	0.5	>8
*M. hyopneumoniae* (18)^b^	Clinafloxacin	≤0.008	≤0.008	≤0.008
	Danofloxacin	0.015–0.03	0.03	0.03
	Enrofloxacin	0.03–0.06	0.06	0.06
*M. bovirhinis* (10)^a^	Clinafloxacin	0.008–0.03	0.008	0.03
	Danofloxacin	0.125	0.125	0.125
	Enrofloxacin	0.125–0.25	0.125	0.25

a Bovine isolates;

b swine isolates.

### TIME-KILL ASSAYS

The results of the time-kill kinetic studies are summarized in **Table 2** when tested at drug concentrations that were four times the MIC. The data are presented in terms of the log CFU change in which cidality is defined as a ≥3.0 log CFU reduction in the initial inoculum at 24 h. All of the test agents produced a similar degree of killing (reduction of 3.8–5.0 log CFU/ml) and were defined as being cidal. The greatest decrease in CFUs occurred between 2 and 6 h for all compounds (data not shown). Clinafloxacin had a log decrease range of 4.4–5.0 against all organisms tested.

**Table 2:** Time-kill results for clinafloxacin and comparator drugs tested at four times the MIC.

Organism	Log decrease (increase) at endpoint for antibiotic ^a^			
	Clina-floxacin	Dano-floxacin	Enro-floxacin	Control
*M. haemolytica* ATCC 33396	4.7	4.1	4.0	(2.2)
AHDRCC 39227	4.8	4.2	3.9	(2.3)
AHDRCC 39228	4.8	4.2	4.0	(2.7)
*P. multocida* ATCC 43137	4.8	4.2	4.0	(2.3)
AHDRCC 39571 (cattle)	4.5	4.6	4.4	(3.3)
AHDRCC 44344 (swine)	4.6	4.4	4.4	(2.7)
*H. somni* ATCC 700025	4.4	3.8	4.3	(2.9)
AHDRCC 38925	4.6	4.0	4.1	(2.0)
*A. pleuropneumoniae*	4.6	4.0	4.2	(2.3)
ATCC 27090				
AHDRCC 44672	4.8	4.2	4.2	(2.4)

a The log CFU per milliliter reduction was calculated at 24 h for all cultures.

### MPC DETERMINATIONS

Determination of MPC/MIC ratios was determined for test compounds using one strain of *S. suis* and *P. multocida* (**Table 3**). For clinafloxacin and *S. suis*, the parent MIC shifted from 0.125 to 0.25 μg/ml for a MPC/MIC ratio of 2 whereas the MIC did not shift with *P. multocida* resulting in a ratio of 1. MPC/MIC ratios using *S. suis* were 16 and 8 for danofloxacin and enrofloxacin, respectively; using *P. multocida* the ratio was 4 for danofloxacin and enrofloxacin.

**Table 3:** Mutant prevention concentration determinations of clinafloxacin and comparator drugs.

Isolate	Drug used for mutant selection	Parent MIC (μg/ml)	MPC (μg/ml)	MPC/MIC ratio
*S. suis* AHDRCC	Clinafloxacin	0.125	0.25	2
43581 (swine)	Danofloxacin	4	64	16
	Enrofloxacin	1	8	8
*P. multocida*	Clinafloxacin	0.015	0.015	1
AHDRCC 44349	Danofloxacin	0.015	0.06	4
(bovine)	Enrofloxacin	0.015	0.06	4

### MOUSE EFFICACY ASSAYS

Clinafloxacin and comparator compounds were efficacious with low ED_50_ values when tested against livestock pathogens in mouse IP or IN models (**Table 4**). ED_50_ values for clinafloxacin were determined when dosed three times at 1, 24, and 48 h and 1 and 3 h after an IP challenge with a bovine strain of *M. haemolytica* (0.019 and 0.008 mg/kg, respectively) while an ED_50_ value of 0.55 mg/kg was obtained when bovine *P. multocida* was administered intranasally and 0.08 mg/kg when administered intraperitoneally. The ED_50_ value was slightly higher at 0.7 mg/kg with the IP *A. pleuropneumoniae* model. No evidence of hypoglycemia or photosensitivity was observed in any mice during these efficacy studies.

**Table 4:** Murine drug efficacy assays.

*In vivo* model (route of infection)	Drugs dosed at:	Mean ED_50_ value (mg/kg/dose)		
		Ceftiofur (*n*)	Clinafloxacin (*n*)	Enrofloxacin (*n*)
Bovine *M. haemolytica* (IP)	1, 24, and 48 h post-challenge	0.28 (5)	0.019 (5)	0.29 (5)
	1 and 3 h post-challenge	0.22 (2)	0.008 (2)	na
Bovine *P. multocida* (IN)	18 h post-challenge	1.05 (2)	0. 55 (2)	na
Bovine *P. multocida* (IP)	1 and 3 h post-challenge	0.11 (3)	0.08 (3)	0.26 (3)
Swine* A. pleuropneumoniae* (IP)	1, 24, and 48 h post-challenge	8.2 (3)	0.7 (3)	1.8 (3)

*IP, intraperitoneal; IN, intranasal; n, number of tests; na, not available.*

## DISCUSSION

The fluoroquinolone antimicrobial agents danofloxacin, enrofloxacin, and marbofloxacin are currently approved for the treatment of BRD and SRD caused by specific bacterial respiratory pathogens. Bacterial resistance is of increasing importance in livestock respiratory diseases while the greatest threat to successful infectious disease treatment and the impetus behind the search for new antibiotics and other treatment modalities are the evolution and spread of antibiotic resistance.

Pan and Fisher (1998) investigated the response of *S. pneumoniae* to clinafloxacin and reported that mutations in neither *gyrA* nor *parC* alone had much effect on activity. In that study, it was determined that high-level resistance to clinafloxacin required four steps to increase MICs from 0.25 to 64 μg/ml and that the four steps to resistance involved sequential mutations in *gyrA* or *gyrB*, in *parC*, in *gyrA* and finally in *parC* or *parE*. The dual targeting of DNA gyrase and topoisomerase IV and the intrinsic potency of clinafloxacin are desirable features in limiting the emergence of bacterial resistance (Piddock, 1994; Jones et al., 1999). Because of clinafloxacin’s unique mechanism of action and its potential to contribute little to the increase and spread of resistant bacteria, this fluoroquinolone antimicrobial was investigated as a potential alternative antibacterial agent for respiratory disease in livestock animals by determining the *in vitro* activity and rodent efficacy when tested against pathogens associated with BRD and SRD.

A recent article by Portis et al. (2012) showed that the *in vitro* susceptibility of *M. haemolytica* respiratory pathogens from 2004 to 2009 has decreased from 89 to 81% with danofloxacin and 95 to 80% with enrofloxacin while the *in vitro* susceptibility of *P. multocida* and *H. somni* respiratory pathogens decreased from 100 to 91 and 100 to 86.2%, respectively, with enrofloxacin. However, an earlier article by Meunier et al. (2004) showed that marbofloxacin was active against 95.4% of bovine pathogenic respiratory strains at the end of a 7-year surveillance period. Numerous articles have attributed decreasing fluoroquinolone susceptibilities to transferable plasmids, mutations in *gyrA*, *gyrB*, *parC*, and *parE*, and efflux as mechanisms of fluoroquinolone resistance (Martinez-Martinez et al., 1998; Jones et al., 2000; Alvarez et al., 2008). The data presented in this study show that MIC_90_ values for clinafloxacin are lower for all relevant pathogenic species versus currently marketed animal health fluoroquinolones used to treat livestock infections. Additionally, this study showed that clinafloxacin cidality is similar to existing fluoroquinolones used to treat livestock infections and has a better MPC/MIC ratio profile.

The MIC data and the unique equipotent dual mechanism of action suggest that development of bacterial resistance for clinafloxacin could be low. This study did not investigate the mechanism of action of clinafloxacin in livestock pathogens, especially the Gram-negative organisms which are the prominent organisms found in livestock respiratory disease. However, it was reported by Piddock et al. (1998) that none of the newer fluoroquinolones tested during the study, except for clinafloxacin with its dual mechanism of action, had improved activities compared to ciprofloxacin for the Gram-negative *P. aeruginosa* and *H. influenzae* isolates. In that study, all second-step mutants, except for clinafloxacin, required 2 μg/ml or more of a fluoroquinolone for inhibition (Piddock et al., 1998).

*In vitro* and *in vivo* results from this study suggest the potential of clinafloxacin as a treatment option for livestock respiratory infections caused by a variety of frequently encountered bacterial species. Clinafloxacin shows good *in vitro* activity and efficacy in mouse models and may be a novel alternative for the treatment of respiratory disease in cattle and pigs with a potential for low resistance development. Further developmental studies with this fluoroquinolone are warranted.

## Conflict of Interest Statement

The authors declare that the research was conducted in the absence of any commercial or financial relationships that could be construed as a potential conflict of interest.
